# How Do We Perceive “Aliens”? About the Implicit Processes Underlying the Perception of People With Alien Paraphernalia

**DOI:** 10.3389/fpsyg.2019.01551

**Published:** 2019-07-03

**Authors:** Nils Brandenstein, Fabian Gebauer, Claus-Christian Carbon

**Affiliations:** ^1^University of Heidelberg, Heidelberg, Germany; ^2^Department of General Psychology and Methodology, University of Bamberg, Bamberg, Germany; ^3^Bamberg Graduate School of Affective and Cognitive Sciences, Bamberg, Germany

**Keywords:** perception, aliens, personality trait, social attitude, automatic processes

## Abstract

People often draw trait assessments of unfamiliar persons on the basis of minimal visual information like facial features. Most studies focus on explicit person evaluations, even though automatic processes of perception are the underlying basis. Furthermore, previous experiments on automatic processes only address very general levels of association. We conducted two experiments employing the multidimensional IAT (md-IAT) to examine automatic processes of perception in a more differentiated way, testing essential variables that are often used to characterize aliens. Results show that personality trait associations of people perceived and categorized as aliens (acquired solely through usage of paraphernalia) are not consistently negative in comparison to more familiar-looking people but might point to the core variables of xenophobic stereotypes (e.g., being aggressive, threatening, and untrustworthy). Proceeding in revealing such variables and testing them might help to understand the main cognito-emotive pattern behind xenophobia and help challenging and tackling stereotypes against aliens.

## Introduction

In our everyday life, we automatically judge foreign people based on very limited visual information. Not only do we determine biological and social categories like age, sex, and morphological group at first sight (Bruce and Young, 2013), we also make direct assumptions about their personality traits and their characters (Oosterhof and Todorov, 2008). The created impression grows rapidly and easily. Exposure times of as little as 100 ms seem to be sufficient for people to build up personality assessments of unfamiliar persons which are often quite reliable and consistent across different persons (Willis and Todorov, 2006). Once a specific view of a person is formed it is very hard to ignore or forget it, even if the assessment is not accurate (e.g., Carney et al., 2007).

What cues are taken into account for a certain perception and how does it form in particular? Bar et al. (2006) argued that the main information gathered at first sight is obviously of a plain perceptual nature, like facial attractiveness (Carbon et al., 2018), facial features (Carbon, 2011), facial expression (Derntl et al., 2009) or facial paraphernalia such as headwear or beards. These are reference points we all quickly draw assumptions about personality traits from (Willis and Todorov, 2006). If this perceptive process is so rapid and intuitive, do people even know the basis on which their image is formed? Fazio (2007) has shown that the accessibility of cognitive processes mediating object (person, respectively) evaluations are often not accessible to the single person; sometimes they just depend on the visual composition of contextual information, e.g., how similar the people are to the one being evaluated (Harsányi and Carbon, 2015). At this point, we are entering the field of more implicit cognitions that people are mostly not aware of. Implicit cognitions are processes people are unable to access and express via introspection, even if they do not hide their thoughts intentionally, e.g., due to social desirability (Nosek et al., 2007). We can certainly ask people about their evaluation of a person and measure behavioral outcomes, but we still do not know about the underlying, implicit cognitions forming them in the first place. Therefore, focusing on these automatic processes seems more promising, if we want to understand the nature of how people are perceived and categorized more deeply. A series of papers have examined the validity and deriving consequences of implicit processes. A meta-analysis by Hofmann et al. (2005) reported an average population correlation of Schmidt-Hunter’s ρ = 0.24 of automatic processes and explicit self-report measures. Moreover, behavioral outcomes such as intergroup behavior can be predicted by automatic processes more validly than through self-report measures (Greenwald et al., 2009). Accordingly, automatic processes seem to not only be the theoretical underlying basis of explicit decisions and behavior, they can also predict them to a certain degree. It is, however, important to note that successful prediction does not mean being able to validly draw causal inferences, as only an experimental design can test causality (Fiedler et al., 2006).

One influential and very widely utilized procedure to address automatic associative processes was introduced by Greenwald et al. (1998). Their so-called *Implicit Association Test (IAT)* was published as a method for assessing individual differences in social cognition by measuring “implicit associations” – a nowadays rather disputed qualification; actually the sheer term “implicit” might already be incorrect and should be replaced by “automatic,” following critical issues by a series of authors, among them prominently Fiedler et al. (2006).

So far, the IAT has been employed in a variety of studies about decision making as well as in social and political psychology. In one of the first IAT studies, Rudman et al. (1999) revealed a faster implicit association of people being more unfamiliar to participants (another religious group, respectively) with the negative attribute dimension “unpleasant.” This general pattern of results was replicated concerning other social (Teachman and Brownell, 2001; Rowatt et al., 2005) or ethnic groups (McConnell and Leibold, 2001; Agerstrom and Rooth, 2009). Overall, people seem to implicitly associate people from whom they seem to be different in certain categories with negative attributes more quickly. As the reported studies indicate, the most frequently investigated evaluations are those in which people are confronted with other people who somehow qualify as alien to them (e.g., other culture, facial features, outer appearance, …). For the sake of simplicity, uniformity and precision we will use the acronym PaCA (“Perceived and Categorized Alien”) to refer to and describe people who qualify as aliens to an observer in any form. On the other hand, people who are not categorized as aliens by observers will be referred to as Non-PaCA.

Despite the multitude of studies in this area, research on the perception of PaCA has not yet dived deeper into the complexity of the underlying automatic processes which might be the basis for negative stereotypes: The majority of studies focus on one dimension only, mainly regarding valence (e.g., positive vs. negative), creating a simple “black and white” perspective which neither helps to understand the phenomenon of negative labeling in a differentiated way nor allows for specifically tackling the problem of negative stereotypes against PaCA. As the original IAT does not account for a multidimensional testing of implicit processes, Gattol et al. (2011) extended this method. The resulting *multidimensional IAT (md-IAT)* enables a broader evaluation through the testing of more than just one attribute dimension. From a structural point of view, this method employs a series of single IATs and is used to measure associations in a more detailed way than a single IAT. The md-IAT was tested regarding its psychometric criteria and it was shown that it is a reliable, valid and sensitive indirect measure of associations (Gattol et al., 2011). Therefore, the md-IAT is more than just a series of single IATs. The new method already lead to the discovery of multidimensional implicit processes in other fields of research, such as perception of car brands (Gattol et al., 2011) or the preference of symmetrical over random patterns (Bertamini et al., 2013). Transferred to the present issue, we used the md-IAT to investigate multidimensional automatic processes, underlying the perception of PaCA, as it is a qualified method for differentially analyzing different facets of automatic associations. This helps to reveal the underlying dimensions in regard to the perception of PaCA instead of just obtaining simple positive/negative evaluations which do not uncover the quality of associations in detail. Although the (md)-IAT was criticized regarding its capability of testing and correctly classifying attitudes and stereotypes (Fiedler et al., 2006), it is well suitable for the present research question of revealing automatic, perceptive associations of attributes to what is stored in the perceivers mind. Furthermore, other methods of measuring implicit (automatic) associations and attitudes (e.g., affective priming) seem to be neither necessarily superior to the (md)-IAT in terms of psychometric criteria (Znanewitz et al., 2018), nor to be the better choice for the present research question, as we are interested in differences in associations across dimensions.

Therefore, the major aim of the present study is to examine how PaCA (operationalized solely through the usage of facial paraphernalia) is processed in terms of automatic processes. To address this question, two studies employing the md-IAT were conducted, Study 1 with four and Study 2 with six attribute dimensions that are typically related to negative stereotypes about aliens.

## Study 1

### Methods

#### Participants

The required sample size was calculated beforehand using the G^∗^Power software program provided by Faul et al. (2007). Based on findings by Gattol et al. (2011) we assumed an effect size of *d* = 0.50 which is qualified as a medium-large effect according to Cohen (1988) for detecting possible differences between at least two attribute dimensions. Setting α = 0.05 and test power 1-β = 0.95 leads to a calculated sample size of *N* = 54. We recruited 60 volunteers for the present study, predicting a potential loss of data for 10% of the participants due to the relatively complex apparatus and procedure we utilized. The majority of the sample consisted of students of (B.Sc.) Psychology at the University of Bamberg, with a majority having grown up in the Bamberg area (Bavaria, Germany). Due to a measuring error (IAT procedure failed to capture the reaction times on several trials), two female subjects had to be excluded from the analysis, yielding a final sample of 58 participants (45 female; *M*_age_ = 22.1 years, *SD* = 2.74). During a debriefing interview, 63.8% reported to have a Christian faith, 32.8% had no religious faith and only one person reported having originally been from an Islamic religious background but claimed to have no devoted faith in Islam.

#### Materials

We used a multidimensional IAT (md-IAT) including four successive IATs. As target attributes we employed the following four bipolar dimensions: (1) peaceful-aggressive (2) safe-unsafe (3) good-bad (4) trustworthy-untrustworthy. Each of the four dimensions included six associated words for every category (for an overview see Table 1). The corresponding, original German wordings are enclosed in parentheses.

**Table 1:** Attribute dimensions and attributes used in the md-IAT with original German wording in parentheses (Study 1).

Attribute dimensions	Positive attributes	Negative attributes
(1) peaceful – aggressive	love (Liebe)	war (Krieg)
	mercy (Gnade)	hate (Hass)
	forgiveness (Vergebung)	attack (Angriff)
	gentle (sanft)	violent (gewalttätig)
	help (Hilfe)	hostile (feindselig)
	gracious (gütig)	cruel (grausam)
(2) safe – unsafe	harmless (harmlos)	deadly (tödlich)
	safe (sicher)	malicious (bösartig)
	cuddly toy (Kuscheltier)	poisonous (giftig)
	baby (Baby)	threatening (bedrohlich)
	flower (Blume)	murder (Mörder)
	feather (Feder)	knife (Messer)
(3) good – bad	wonderful (wundervoll	terrible (schrecklich)
	happiness (Freude)	torture (Qual)
	luck (Glück)	sorrow (Leid)
	success (Erfolg)	failure (Misserfolg)
	gift (Vergnügen)	vicious (übel)
	smile (Lächeln)	evil (böse)
(4) trustworthy – untrustworthy	honest (ehrlich)	dishonest (unehrlich)
	upright (aufrecht)	fraudulent (verlogen)
	truthful (wahrhaftig)	insincere (falsch)
	obliging (hilfsbereit)	untruthful (unglaubwürdig)
	family (Familie)	cheeky (dreist)
	friend (Freund)	fraud (Betrüger)


The target concepts consisted of two groups of images: Facial images of people with the typical attributes of Western Christian people (target concept *Christian*, Non-PaCA) and facial images of people with the typical attributes of alien people, perceived and categorized as Muslims (target concept Muslim, PaCA). Each concept consisted of six true color high-resolution portrait photographs of exclusively male people supposed to be “Christians” (Non-PaCA) or “Muslims” (PaCA), respectively. People who acted as photographic models were in fact the same for the PaCA and Non-PaCA face sets – the only difference between both categories being the usage of facial paraphernalia; facial in terms of paraphernalia which are displayed in the face area such as a long beard or a turban. As depicting stereotypical “Christians” (Non-PaCA) is comparatively more difficult, this group mainly consisted of white, middle aged males with rather short hair and a clean shave. These faces served as a basis to create images of PaCA upon. Accordingly, the paraphernalia were just added to impose the alien-specific outward appearance in order to qualify as PaCA, while other facial qualities were preserved to limit the source of variance solely to the paraphernalia employed. Important note: The authors neither want to create the impression that all members of Western Christian groups or Muslim groups wear such paraphernalia, nor that these specific paraphernalia are most specific for these cultural areas. The employment of these paraphernalia merely aims to depict some ingredients that are often shown when depicting people from these cultural areas. Table 2 shows two exemplary versions which were used in the study and which depict the very same person, one for the PaCA and one for the Non-PaCA target group.

**Table 2:** Exemplary faces (plain and modified with paraphernalia) used in the md-IAT (Study 1 and 2).

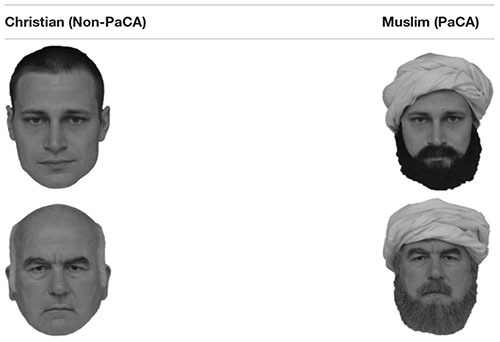

The presentation size of the faces in the md-IAT was 12 cm in height by 9 cm in width with a visual angle of approximately 7° by 5°. All other materials can be received from the corresponding author upon request. To ensure that the set of faces and words really fit the according categories, as suggested in Nosek et al. (2007), all material was rated in a pre-study making sure that the appearance was prototypical for the respective target category and that the images made an authentic impression, resulting in faces that represent PaCA. In this pre-study, ten volunteers (eight male, *M*_age_ = 27.3 years, *SD* = 8.12) rated a set of 25 PaCA versions on a 7-point Likert Scale, regarding the authenticity of the paraphernalia, the prototypicality of being a “Muslim” face and the suitability for the aim of the study. According to participant ratings, six faces with the highest authenticity were selected (*M*s = 5.46, 5.22, 5.31, 5.13, 4.63, and 4.40). This pre-study and the obtained ratings ensure that the employed faces are ecologically valid in regard to qualify as PaCA, so that the later results can be analyzed accordingly. With regard to the terms used for the target attribute categories, we wanted to ensure best possible fits of single terms and category, so we conducted a further pre-study on this issue. Seven volunteers (none having taken part in study 1; all female, *M*_age_ = 20.6 years, *SD* = 0.73) rated 48 terms (adjectives and nouns) in total with regard to their respective unambiguousness and affiliation to the pre-experimentally assigned target attribute categories on a 7-point scale, ranging from 1 = ambiguous to 7 = unambiguous. The mean level of unambiguousness was very high (*M* = 6.16, *SD* = 0.66) indicating very good fits of the categories and the assigned terms, respectively. Prior to the rating, the same seven participants were asked to name three words (adjectives or nouns) which they thought fit best to reflect each category. The most frequently mentioned words replaced the least fitting ones from the mentioned rating: In dimension (2) safe – unsafe, the word “cuddly toy” replaced the word “healing,” “gift” replaced the word “miracle” in dimension (3) good – bad and the word “family” replaced the word “trusty” in dimension (4) trustworthy – untrustworthy. No word was replaced in dimension (1) peaceful – aggressive, as all of the five chosen words in this particular dimension received the highest unambiguity rating possible.

#### Apparatus

The md-IAT was administered using a standalone Java application, programmed at the Department of General Psychology and Methodology. The experiment was run on a Fujitsu Esprimo P700 E90+ Computer (Intel Core i3-2120, 3.30 GHz; 6 GB DDR3 RAM) with preinstalled Windows 7 Professional (V.3.1.4). Participants sat approximately at a distance of 55–60 cm away from the screen – a 24-inch LG Flatron E2411 LED Monitor at a resolution of 1,920 × 1,080 pixels, with a refresh rate of 60 Hz. A self-developed USB Button Box was used as default input device, enabling the measurement of reaction times synchronized by the physical appearance of the stimulus and with a time resolution of ≤1 ms.

#### Procedure and Design

First, participants had to sign a written consent explaining their contribution to the research and their rights in the present experiment, as well as filling out pre-tests regarding visual abilities and handedness. All procedures were in accordance with the Declaration of Helsinki. The study was in full accordance with the ethical guidelines of the University of Bamberg and was approved by an umbrella evaluation of the university ethics committee on August 18, 2017.

Subsequently, the IAT was administered requiring participants to complete four single IATs organized as separated “dimension sessions” – one session for each of the four bipolar attribute dimensions. The order of the dimension sessions was fixed throughout the entire experiment: (1) peaceful-aggressive (2) safe-unsafe (3) trustworthy-untrustworthy (4) good-bad. In each trial, participants were asked to respond to the respective task as quickly and accurately as possible in a two-alternative-forced-choice (2AFC) scheme by pressing the left or the right button on the button box. Incorrect answers were indicated by a subsequently presented red capital “X,” upon which a participant had to press the alternative button. Correct answers were not specifically indicated. All stimuli (faces and words) were fully randomized across participants to avoid possible learning effects even after having responded to a mass of trials. Each dimension session contained five blocks – the first two blocks were practice blocks to train the coordination of responses. In block 1, participants had to categorize the facial images of the target groups “Muslim” (PaCA) and “Christian” (Non-PaCA), by, e.g., pressing the left button for “Muslim” and the right button for “Christian.” In block 2, participants had to decide to which attribute category a certain term fitted. In both of these blocks, 12 trials had to be carried out correctly. Following typical IAT routines, in the subsequent block target categories (faces) were combined with target attributes (terms). For instance, participants had to respond to the combination “Muslim and peaceful” with the left button and “Christian and aggressive” with the right button. After block 3, a practice block (12 trials) was again administered where the sides of the attribute category were reversed so that the left button was assigned to “Christian” and the right button to “Muslim,” respectively. Then in block 5, the combinations of the concepts and attributes were concordantly reversed (e.g., “Christian and peaceful” for the left button and “Muslim and aggressive” for the right button). Both the block 3 and 5 consisted of 24 trials. The reaction times (RTs) of blocks 3 and 5 were used for calculating the strength of automatic associations later on. It must be noted that this procedure omits the two additional practice blocks of the congruent and incongruent task (block 3 and 6), proposed by Greenwald et al. (1998), leading to five instead of seven blocks in total. Although the inclusion of these practice trials can be informative and, e.g., augment the correlation between the IAT score and self-report measures (Greenwald et al., 2003), we excluded them due to the already very intense and potentially exhaustive procedure of the md-IAT to limit fatigue that could have contributed to a potential increase in errors and dropouts.

As mentioned above, each dimension session consisted of five blocks (abbreviated as B1 through B5). The relevant blocks B3 and B5 were in fact separate blocks, but due to the scoring algorithm, the *D-*measure proposed by Greenwald et al. (2003), they were analyzed as joint data by creating a ratio between the response latencies. The *D-*measure is a scoring algorithm that outperforms the previous calculation schemes by addressing most of their problems: (1) Minimizing the correlation between IAT effects and individual response latencies, (2) reducing the effect of the order of the IAT blocks, as well as (3) the effect of previously completing IATs on IAT scores (learning effect). It simultaneously features (4) strong internal consistency, and (5) maximizes the correlation between implicit and explicit measures. Subsequently, the resulting *D-*measures of these two blocks were used as dependent measure. The whole procedure took about 30 min. Finally, all participants were debriefed, and thanked for their participation.

### Results

#### Implicit Measures

As noted earlier, the present analysis of reaction times is based on the scoring algorithm *D-*measure introduced by Greenwald et al. (2003), which divides the reaction time difference of the two combined blocks (B5 and B3) through their joint standard deviation. Gattol et al. (2011) presented another scoring algorithm when using the md-IAT, the *adapted *D-*measure*, relying on a dynamic outlier criterion. Due to similar effect sizes documented by Gattol et al. (2011), we continued using the standard *D-*measure scoring algorithm to allow for a better comparison across studies from research on stereotypes. Accordingly, trials with responses either lower than 300 ms or above 3,000 ms were treated as outliers and were consequently excluded.

**FIGURE 1:**
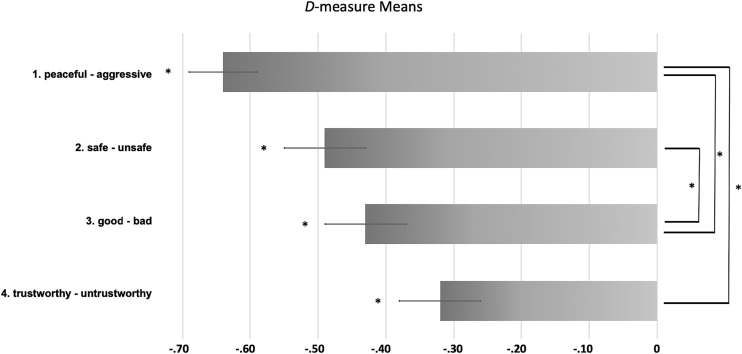
Comparison of mean *D*-measures of the four attribute dimensions. Means are displayed with ±1 standard error of the mean (SEM). Negative *D*-measure scores indicate a faster response ratio to the negative dimension concept and PaCA and the positive dimension concept and Non-PaCA, respectively. *D*-measures significantly differing from zero (Tukey-Test) are marked with ^∗^, indicating *p* < 0.05.

Table 3 lists the results of all four IATs, providing the *D-*measure, standard errors and *t*-test statistics. A repeated-measures ANOVA with the within-subjects variable attribute dimension was calculated, to test for simple main effects of the different IAT dimensions. Mauchly’s Test of sphericity showed no violation of the sphericity assumption, as did a test for normal distribution of the dependent variables. A significant main effect of attribute dimension was observed, *F*(3,171) = 10.52, *p* < 0.001, η_p_^2^ = 0.156. Due to this effect, a *post hoc* *Tukey Test* was administered to reveal differing attribute dimensions. Results showed that the dimensions (1) peaceful-aggressive – (3) trustworthy-untrustworthy (*p* < 0.001), (1) peaceful-aggressive – (4) good-bad (*p* < 0.01) and (2) safe-unsafe – (3) trustworthy-untrustworthy (*p* < 0.05) differed significantly. Moreover, *t*-Tests for all *D-*measures of the attribute dimensions showed a statistical difference from zero (all *p* < 0.001, see Table 3). The mean *D-*measures of all six attribute dimensions, *t*-Tests from zero and significantly differing pairs are presented in Figure 1. Participants religious denomination did not affect any of the reported results: The repeated-measures ANOVA model was also calculated as a mixed linear model (equivalent to a mixed ANOVA) including participant’s faith (faith vs. no faith) as a fixed effect (between-participants factor, respectively) which failed to reach statistical significance on the *D*-measures.

**Table 3:** Mean *D*-measures, standard error (*SE*) and *t*-test statistics of all four IATs.

Attribute dimension	*N*	*D-measure*	*SE*	*t*	*df*	*P*
(1) peaceful – aggressive	59	–0.64	0.05	–12.66	58	< 0.001^∗∗^
(2) safe – unsafe	59	–0.49	0.06	–8.97	58	< 0.001^∗∗^
(3) good – bad	59	–0.43	0.06	–4.92	58	< 0.001^∗∗^
(4) trustworthy – untrustworthy	59	–0.32	0.06	–7.63	58	< 0.001^∗∗^


*Negative *D*-measure scores indicate a response latency ratio of faster overall response to the negative dimension concept and PaCA and the positive dimension concept and Non-PaCA, respectively. Significantly differing attribute dimensions from zero are indicated by ^∗∗^*p* < 0.001.*

## Study 2

Study 2 was intended to replicate the findings of Study 1 with methodological improvements and extensions. The category pairs (5) worthy-worthless and (6) productive – lazy served as additional dimensions in the md-IAT. Furthermore, the order of dimension presentation was counterbalanced throughout the participants. All stimuli materials were rated in a pre-study to ensure an unambiguous fit with their designated dimension category.

### Methods

#### Participants

For the second study, 37 persons participated. Due to the high effect size observed in Study 1, the number of participants ought to be sufficient for the present analysis. The recruiting process included advertising at the Institute of Psychology in Bamberg, as well as posting in several regional online forums. Age range varied from 18 to 51 years. Due to a measuring error, two participants had to be excluded from the analysis, leaving 35 participants (24 female, *M*_age_ = 26.4 years, *SD* = 7.4). During the debriefing interview, 65% of the sample reported Christian faith and 35% reported no faith.

#### Materials and Apparatus

As in Study 1, we used a md-IAT with six successive IATs. Again, the target concepts were “Muslim” (PaCA) and “Christian” (Non-PaCA) with the following six bipolar dimension sessions: (1) peaceful-aggressive (2) safe-unsafe (3) good-bad (4) trustworthy-untrustworthy (5) worthy-worthless and (6) productive-lazy. Each dimension included six faces of PaCA and Non-PaCA faces (identical to those in Study 1) and five words (adjectives), which were again rated prior to use in the md-IAT (see Tables 2, 4 for an overview of the used stimuli). The corresponding, original German wordings are enclosed in parentheses. We conducted a Binomial Test for all words, based on a free matching task of the words and dimensions with 15 people (9 female, *M*_age_ = 39.4 years, *SD* = 16.5). Every word used in Study 2 has been significantly categorized with its designated dimension (all *p*_s_ < 0.05). Due to this matching method, the attributes used in Study 2 slightly deviate from those in Study 1 but are nonetheless unambiguously associated with their respective dimensions, just like the attributes used in Study 1.

**Table 4:** Attribute dimensions and attributes used in the md-IAT with original German wording in parentheses (Study 2).

Attribute dimensions	Positive attributes	Negative attribute
(1) peaceful – aggressive	gentle (sanft)	hostile (feindselig)
	peaceful (friedlich)	aggressive (aggressiv)
	affectionate (liebevoll)	hate-filled (hasserfüllt)
	calm (ruhig)	militant (kriegerisch)
	favorable (wohlwollend)	violent (gewalttätig)
(2) safe – unsafe	harmless (harmlos)	deadly (tödlich)
	non-dangerous (ungefährlich)	dangerous (gefährlich)
	unthreatening (unbedrohlich)	threatening (bedrohlich)
	safe (sicher)	unsafe (unsicher)
	benign (gutartig)	malicious (bösartig)
(3) good – bad	friendly (freundlich)	unfriendly (unfreundlich)
	kind (nett)	mean (gemein)
	polite (höflich)	impolite (unhöflich)
	pleasant (angenehm)	unpleasant (unangenehm)
	good (gut)	bad (schlecht)
(4) trustworthy – untrustworthy	honest (ehrlich)	dishonest (unehrlich)
	upright (aufrecht)	false (verlogen)
	truthful (wahrhaftig)	untruthful (falsch)
	credible (glaubwürdig)	unreliable (unglaubwürdig)
	trustworthy	untrustworthy (nicht-
	(vertrauenswürdig)	vertrauenswürdig)
(5) worthy – worthless	worthy (wertvoll)	worthless (wertlos)
	precious (kostbar)	inferior (minderwertig)
	useful (nützlich)	useless (nutzlos)
	irreplaceable (unersetzbar)	replaceable (überflüssig)
	extraordinary (besonders)	ordinary (gewöhnlich)
(6) productive – lazy	effective (effektiv)	ineffective (ineffektiv)
	productive (tüchtig)	lazy (faul)
	fast (schnell)	slow (langsam)
	motivated (motiviert)	unmotivated (unmotiviert)
	focused (zielstrebig)	powerless (antriebslos)


#### Procedure and Design

The procedure and design of Study 2 was identical to that of Study 1, except for the two additionally administered IATs and the counterbalancing of the presentation of the dimension sessions throughout the participants to limit possible learning effects even further.

### Results

#### Implicit Measures

**FIGURE 2:**
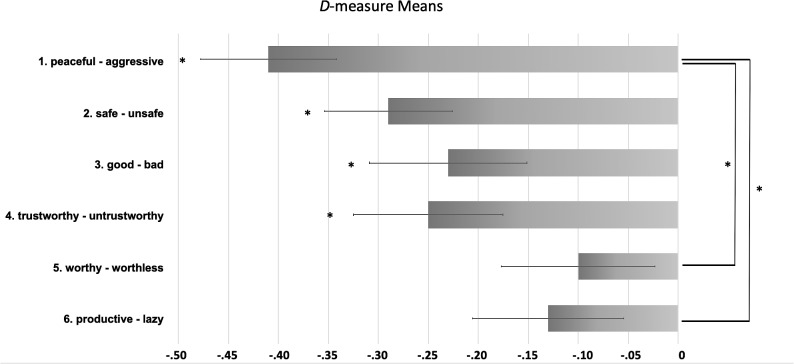
Comparison of mean *D*-measures of the six attribute dimensions. Means are displayed with ±1 standard error of the mean (SEM). Negative *D*-measure scores indicate a faster response ratio to the negative dimension concept and PaCA and the positive dimension concept and Non-PaCA, respectively. *D*-measures significantly differing from zero (Tukey-Test) are marked ^∗^*p* < 0.05.

Study 2 utilized the same algorithm (*D*-measure) for analyzing and comparing the response latencies as Study 1. Table 5 demonstrates the results of the six conducted IATs with the coherent *D*-measures, standard errors and *t*-test statistics. Similar to Study 1, all *D*-measures were negative, indicating a faster response with the negative dimension concept and PaCA and vice versa for Non-PaCA. A repeated-measures ANOVA with the within-subjects variable attribute dimension revealed a significant simple main effect for attribute dimension, *F*(5, 170) = 3.24, *p* < 0.01, η_p_^2^ = 0.087, with no violation of sphericity or normal distribution. To detect the significantly differing dimensions, a *post hoc* *Tukey Test* was again administered. The attribute dimension (1) peaceful-aggressive differed from the two attribute dimensions (4) worthy-worthless (*p* < 0.01), and (6) productive-lazy (*p* < 0.05). *T*-tests for all *D*-measures of the attribute dimensions showed a statistical difference from zero, except for dimension (5) worthy – worthless and dimension (6) productive –lazy. The mean *D*-measures of all six attribute dimensions, *t*-tests from zero and significantly differing pairs are presented in Figure 2. As in Study 1, the faith did not affect any of the final results (tested in a mixed linear model including religious denomination as fixed effect).

**Table 5:** Mean *D*-measures, standard error (*SE*), and *t*-test statistics of all six IATs.

Attribute dimension	*N*	*D-measure*	*SE*	*t*	*df*	*p*
(1) peaceful – aggressive	35	–0.41	0.07	–6.07	34	< 0.001^∗∗^
(2) safe – unsafe	35	–0.29	0.06	–4.57	34	< 0.001^∗∗^
(3) good – bad	35	–0.23	0.08	–2.92	34	0.006^∗^
(4) trustworthy – untrustworthy	35	–0.25	0.07	–3.37	34	0.002^∗^
(5) worthy – worthless	35	–0.10	0.08	–1.32	34	0.195
(6) productive – lazy	35	–0.13	0.08	–1.75	34	0.090


*Negative *D*-measure scores indicate a response latency ratio of faster overall response to the negative dimension concept and PaCA and the positive dimension concept and Non-PaCA, respectively. *D*-measures significantly differing from zero are indicated ^∗∗^*p* < 0.001, ^∗^*p* < 0.05.*

## General Discussion

The aim of the present experiments was to investigate the nature of automatic processes underlying the perception of alien people (PaCA), acquired through use of paraphernalia. Until now, research on automatic processes has only accounted for a unidimensional method of evaluation and typically found PaCA to be automatically associated with more negative attributes. However, it was hypothesized that PaCA is not always associated with negative attributes; it rather depends on the specific attribute dimension. Therefore, we used the md-IAT to investigate the nature and different facets of automatic associations, as this method is – despite the reasonable criticism mentioned in the introduction – capable of revealing differences in associations or how “closely” related a given attribute dimension is to what has been stored in memory, respectively. The results of Study 1 and Study 2 provide strong evidence for the hypothesis of manifold associations: Both administered md-IATs revealed a multidimensionality in the automatic processes of perception, contingent on which specific attribute we look at. This implies that the perceptive “black and white” image regarding PaCA in most studies underestimates the complexity of the underlying automatic processes. The individual reaction time ratios (*D*-measures) also demonstrate this complexity: Even though all *D*-measures were negative, indicating a general tendency to associate PaCA quicker with negative attributes, *D*-measures of the two dimensions (5) worthy – worthless and (6) productive – lazy in Study 2 did not reach statistical significance from zero, showing that in these particular dimensions, perception of PaCA and Non-PaCA are comparable. On the other hand, the other dimensions in both experiments delivered typical findings: PaCA were much more quickly associated with negative than positive attributes and vice versa for Non-PaCA.

As our perception of people is naturally neither completely positive nor negative, it is deemed valid that the conducted studies revealed this pattern of results. In our case, for instance, attributes like “worthless” or “lazy” are not typically implicit profiles in regard to PaCA and – on the other hand – profiles like, for instance, “aggressive” are. Oosterhof and Todorov (2008) argued that face evaluations serve as an adaptive mechanism for inferring harmful intentions. This could explain the quicker association of PaCA with the negative attributes “aggressive,” “unsafe,” “bad,” and “untrustworthy,” as they are regarded as harmful traits. On the other hand, attributes like “worthy” – “worthless” and “productive” – “lazy” do not fall into an evaluation of a person’s possible menace. Subsequently, participants did not show a perceptive tendency of either group. Another explanation could lie in the initial formation of associations: One of the most prominent influencing factors of association and stereotype formation are learning effects in the social context (e.g., learning from role models, significant others) that contribute to the formation – and manifestation over time – of assumptions and associations with PaCA, respectively (Bar-Tal, 1997). It could be assumed that the identified core variables, associated with PaCA in Study 1 and 2, occur more frequently in the person’s social environment than attributes like “worthless” or “lazy,” which then results in the observed perceptive tendencies. Setting these discrepancies in the perceptive dimensions into the context of the “Stereotype Content Model” (SCM) by Cuddy et al. (2009), our results fit the model’s prediction fairly well: In general, the SCM proposes potentially universal principles of societal stereotypes and their relation to social structure. The authors stated that “(…) many groups are tagged as proficient in one sphere (i.e., either warmth or competence) and inferior in the other” (p. 3). The PaCA group was not perceived as lazier or less proficient than the Non-PaCA group, but was perceived as less “warm,” leading to the perception of PaCA being untrustworthy, bad, aggressive and unsafe.

Comparing the results of both experiments, we see that Study 1 delivered more significantly differing dimension pairs than Study 2 did. A possible explanation could be a smaller sample size, as we expected high effect sizes after inspecting the results of the first study, so the same dimension pairs (as in Study 1) did not reach significance in Study 2. Actually, the general size of all *D*-measure scores turned out to be lower in Study 2 as well, although they still significantly differed from zero. As the order of dimension sessions was counterbalanced in Study 2 (adjustment of method), a possible learning/order effect could also contribute to more differing attribute dimensions in Study 1. Although this effect could potentially account for the differing dimensions in Study 1, it cannot account for the arguably very similar dimension effect in Study 2. Therefore, a sheer order effect cannot account for the obtained resulting pattern of data.

Setting all into context, the pure existence of paraphernalia is enough to trigger automatic processes of perception, which are – at least partly – different to Non-PaCA. Even though our perception of PaCA is not uniformly biased, as the findings of the present studies suggest, we still tend to automatically associate PaCA with negative, harmful attributes more frequently. Due to this broader understanding of perception, we are able to make better predictions on how people will estimate PaCA or interact with them. We will discuss our inference of the obtained results, possible caveats and confounding factors in the following. Even though sample size was calculated beforehand, a higher sample size would be desirable to really make generalization assumptions to the underlying population. A higher sample size would then also allow for other statistical methods of testing multidimensionality, like, e.g., exploratory or even confirmatory factor analysis. However, due to the complexity and effort of the md-IAT, realizing high *N*s is very resource intensive, as already noted by Carbon (2018). Our samples were dominated by female participants (78% female in Study 1, 69% female in Study 2). This numerical disparity could limit the generalizability of the results as well, as a growing body of research has reported gender differences in prejudice and stereotypes (e.g., Rudman and Goodwin, 2004). However, these differences were found in studies that deliberately activated the psychosocial gender roles in participants’ minds, as they were used as the target concepts. In our studies, the activation of participants’ religious group is more likely to have happened and differences in gender regarding the perception of PaCA/Non-PaCA don’t seem reasonable in this scenario. Nevertheless, the potential influence of confounding factors like participants’ gender should be addressed in upcoming studies regarding the perception of PaCA. When conducting the experiment in non-western societies it would also be highly interesting to see, if the obtained pattern of results would e.g., be reversed in a Muslim-coined society. This is due to the fact that “strangeness” of faces is always inherently biased by the societal context, experience and also religion. Broadly spoken, we are more familiar with people that we encounter, interact with and that are referred to in our society, which influences our mindset on what we see as PaCA or Non-PaCA. When investigating the associations with PaCA/Non-PaCA, we will certainly have to use different visual cues for each society to make sure that the faces (or stimuli in general) portray stereotypical characteristics of the particular groups. It was brought to our attention that veiling of faces can automatically lead to a higher perception of thread and the results are not due to the alien characteristics of the faces. While this seems to be true for obfuscation that affects important facial areas to infer traits and emotions from, like the mouth or eye area (Fischer et al., 2012), the paraphernalia added in our studies don’t meet these criteria. All facial cues to make inferences from are still visible and the turban and beard are more likely to indicate the affiliation to another social, ethnic, or religious group (e.g., Unkelbach et al., 2008). Nevertheless, future research will be required to evaluate the facets of automatic processes even more. Thereby, we will be able to better understand the cognito-emotive pattern behind xenophobic stereotypes and can start to resolve them, as described in our following conclusion.

## Conclusion

In another paper on racial categorization (Harsányi and Carbon, 2015) we called into attention what Guillaumin (1999) stated about the categorization and often-supposed-but-not-verified differences between people of different origin: “Race does not exist. But it does kill people” (p. 46). In the present paper we revealed that the very same person, when depicted with or without paraphernalia which are typically associated with Muslim culture, is not only perceived as Muslim vs. Christian but is also linked with pejorative associations in the case of Muslim framing. By employing the multidimensional IAT – md-IAT (Gattol et al., 2011) we were able to further differentiate which kind of personality characteristics perceivers associated with such depictions. Beside associating the respective persons with such more unspecific qualities like “bad” the perceivers also associated more specific qualities like “unsafe,” “aggressive,” and “untrustworthy” – all in all very negative associations which do not invite open dialogues, meetings and mutual exchange of personal and cultural knowledge. However, exactly these means are essential for better knowledge of each other, an alteration of biased associations, as well as the enabling of peaceful co-existence, as stated in the Intergroup-Contact-Theory (Pettigrew, 1998) and shown in many studies investigating the effect of direct contact and interaction with outgroup members (Pettigrew and Tropp, 2006; Galli et al., 2015). How to address these issues? Associations are initiated and manifested by role models, learning, and everyday routines and practices (Hamilton and Gifford, 1976; Bar-Tal, 1997). If we work on all these facets of life, concretely, (1) if we frequently refer to positive role models and make their stories public, (2) if we learn how others live, how they share beliefs, how common everyday practices are and engage with them, and finally (3) if we better check our language usage and our behavior with seemingly alien persons, we have the chance of establishing a new, less stereotypically shaped picture of people of different origin, based on individual and real facts.

## Data Availability

All the files, scripts, and datasets are also available in OSF: https://osf.io/x8jwk/.

## Ethics Statement

All procedures were in accordance with the Declaration of Helsinki. The study was in full accordance with the ethical guidelines of the University of Bamberg and was approved by an umbrella evaluation of the university ethics committee on August 18, 2017.

## Author Contributions

C-CC had the initial idea. All authors developed the study concept. All authors contributed to the study design. NB performed the testing and data collection. NB and FG performed the data analysis. NB and FG performed the interpretation of the data under the supervision of C-CC. NB drafted the initial manuscript. C-CC provided the critical revisions. All authors approved the final version of the manuscript for submission.

## Conflict of Interest Statement

The authors declare that the research was conducted in the absence of any commercial or financial relationships that could be construed as a potential conflict of interest.
